# Understanding suboptimal insulin use in type 1 and 2 diabetes: a cross-sectional survey of healthcare providers who treat people with diabetes

**DOI:** 10.1186/s12875-024-02390-9

**Published:** 2024-04-22

**Authors:** Rachel S. Newson, Erik Spaepen, Birong Liao, Julie Bower, Indranil Bhattacharya, Esther Artime, William Polonsky

**Affiliations:** 1 Real World Evidence Eli Lilly and Company, 60 Margaret Street, Sydney, NSW 2000 Australia; 2 HaaPACS GmbH, Schriesheim, Germany; 3 Eli Lilly and Company, Indianapolis, IN USA; 4 Eli Lilly and Company, Gurgaon, India; 5 Eli Lilly and Company, Alcobendas, Spain; 6 Behavioral Diabetes Institute, San Diego, CA USA

**Keywords:** Diabetes, Suboptimal insulin dosing, Healthcare providers, Missed doses

## Abstract

**Background:**

The purpose of this study was to understand the healthcare provider (HCP) perspective on the extent of suboptimal insulin dosing in people with diabetes (PwD), as well as specific challenges and solutions to insulin management.

**Methods:**

An online survey of general practitioners and specialists (*N* = 640) who treat PwD in Germany, Spain, the United Kingdom, and the United States was conducted. Responses regarding HCP background and their patients, HCP perceptions of suboptimal insulin use, and challenges associated with optimal insulin use were collected. Categorical summary statistics were presented.

**Results:**

Overall, for type 1 diabetes (T1D) and type 2 diabetes (T2D), most physicians indicated < 30% of PwD missed or skipped a bolus insulin dose in the last 30 days (T1D: 83.0%; T2D: 74.1%). The top 3 reasons (other than skipping a meal) HCPs believed caused the PwD to miss or skip insulin doses included they “forgot,” (bolus: 75.0%; basal: 67.5%) “were too busy/distracted,” (bolus: 58.8%; basal: 48.3%), and “were out of their normal routine” (bolus: 57.8%; basal: 48.6%). HCPs reported similar reasons that they believed caused PwD to mistime insulin doses. Digital technology and improved HCP-PwD communication were potential solutions identified by HCPs to optimize insulin dosing in PwD.

**Conclusions:**

Other studies have shown that PwD frequently experience suboptimal insulin dosing. Conversely, results from this study showed that HCPs believe suboptimal insulin dosing among PwD is limited in frequency. While no direct comparisons were made in this study, this apparent discrepancy could lead to difficulties in HCPs giving PwD the best advice on optimal insulin management. Approaches such as improving the objectivity of dose measurements for both PwD and HCPs may improve associated communications and help reduce suboptimal insulin dosing, thus enhancing treatment outcomes.

**Supplementary Information:**

The online version contains supplementary material available at 10.1186/s12875-024-02390-9.

## Background

Achieving optimal glycemic outcomes is critical for effective management of diabetes, and basal-bolus treatment regimens are currently seen as the standard of care for insulin injection therapy for fasting and post-prandial glucose in people with diabetes (PwD) [1–3]. As these treatment regimens are complex (i.e., multiple injections in a day) and are personalized to account for factors such as lifestyle and food intake, healthcare providers (HCPs) must carefully prescribe treatment regimens based on individual patient needs and ensure adequate self-management education. The complexity of these regimens and lack of time HCPs have to help PwD manage treatment creates challenge [4], often leading to suboptimal insulin management [5].

Support from a comprehensive team to navigate the complexities of diabetes treatment is important for diabetes self-management, and includes, but is not limited to, emotional, physical, nutritional, and psychosocial care [1]. HCPs play a vital role in supporting a patient’s self-management of diabetes treatment regimens [1]. The American Diabetes Association recommends PwD and HCPs work together to optimize management goals and efforts [6], but the majority of insulin treatment optimization falls on self-management, where the patient must ensure they are taking the right amount of insulin at the right times every day.

Anecdotal evidence shows differences between how HCPs and PwD view the extent and cause of suboptimal insulin dosing. As HCPs are responsible for prescribing insulin treatment regimens, taking into account the needs and lifestyles of PwD, understanding these differences in perspective will help identify effective solutions. As such, the objective of the current study was to understand perspectives of HCP on the extent of suboptimal insulin dosing in PwD, as well as specific challenges and solutions to insulin management.

## Methods

### Study design

This study was a cross-sectional, non-interventional, online survey of people with type 1 diabetes (T1D) and/or type 2 diabetes (T2D) and HCPs in Germany, Spain (HCP survey only), the United Kingdom, and the United States (US). Results reported here are pooled across countries for the HCP survey. Results for the patient survey are reported elsewhere [7].

HCPs eligible for enrolment in the study were primary care physicians (i.e., general practitioners) and specialists (i.e., diabetologists/endocrinologists) who were actively working with adult PwD (seeing at least 5 insulin-using PwD per week) and initiating care with non-connected insulin pens. HCPs were also required to have at least 2 years of post-graduation experience. All participants provided informed consent either as part of the online survey for the pilot phase or through an online registration tool for the main study phase. Participants were recruited through a verified panel. All participants were single-blinded to the study sponsor, and the online survey responses were tracked by unique participant identifiers during data collection. Soft quotas for enrolment criteria for each country included 80 primary care physicians and 80 specialists. HCPs eligible for the pilot study were also able to read, speak, and write in English sufficiently well; willing and able to participate in the 45-min online survey and 60-min one-on-one telephone interview; and willing to be audio recorded during the interview.

### Survey

A qualitative pilot phase was conducted to test the content and usability of the US English version of the survey, and participants’ feedback was incorporated into the second, main study survey. The pilot study included 2 general practitioners and 2 specialists (diabetologist and endocrinologist) residing in the US. Participants participated in a 45-min online survey followed by 1 qualitative one-on-one telephone interview up to 60-min. Behavioral questions regarding diabetes, insulin use, and unmet needs, as well as sociodemographic and clinical data, were collected from the survey. During the pilot interview, participants were asked about the clarity, ease of completion, and interpretation of questions. Participants were also asked about the interpretation and appropriateness of response scales, appropriateness of the survey format, and the comprehensiveness of the survey. Ethical approval was provided by the Ethical & Independent Review Service (Study Number 21052–01, approved on 03-Jun-2021).

Country- and language-specific surveys were developed once changes were incorporated into the main US English survey and resubmitted for ethics approval (Study Number 21052-01A, approved on 16-Nov-2021). The final HCP survey consisted of 53 questions, including 7 items for screening/eligibility; 12 items on introducing the survey, informed consent, and current treatment and routines; 19 items on the impression of insulin dosing routines of PwD; and 15 items on insulin dosing issues and solutions. Behavioral questions regarding diabetes, insulin use, and unmet needs as well as sociodemographic and clinical data were collected from the surveys (full survey is provided in Additional File 1).

The main study was conducted from November 2021 through February 2022 and consisted of a 45-min online survey. Participants were instructed to provide their views on insulin usage and dosing and patient self-management of treatment. HCPs were asked to focus on their adult patients with T1D and T2D who use insulin pens. The survey questions were all multiple choice in format, and participants were instructed to select all that applied or select only 1, depending on the question. Participants were required to complete the previous item before moving on to the next question. Missing an insulin dose was defined as any time the PwD did not take a dose that they should have taken, intentionally or unintentionally; and a mistimed insulin dose was defined as an insulin dose taken at the wrong time (not within 10 to 15 min before a meal for bolus/mealtime insulin) or not at the usual time (for basal insulin) (Additional File 1). Participants were compensated for their time in line with fair market value payments based on experience and country legislations.

### Statistical analyses

All analyses were descriptive in format, and categorical variables were summarized as numbers and percentages. Missing data were not possible because the survey was programmed such that a participant could not move on to the next question before completing the previous item. In the event a participant terminated before completing the survey, they were not counted as part of the study population. Statistical analyses were performed using SAS version 9.4 or higher (SAS Institute, Cary, NC).

## Results

### Demographics and clinical characteristics

Overall, 640 HCPs completed the online survey (Table 1). Most HCPs were male (*n* = 435, 68.0%) and were from a Caucasian background (*n* = 435, 68.0%). A total of 592 (92.5%) HCPs had more than 5 years of experience practicing medicine: 50% were specialists and 50% were primary care physicians. A total of 530 (82.8%) HCPs treated more than 10 PwD per week with insulin pens.

**Table 1:** Demographics and clinical characteristics

Characteristic, *n* (%)	Total (*N* = 640)
Female	196 (30.6)
Race/ethnicity	
White	435 (68.0)
Black or African American	18 (2.8)
Asian	121 (18.9)
Hispanic/Latino	31 (4.8)
Native Hawaiian or other Pacific Islander	1 (0.2)
American Indian and Alaskan Native	1 (0.2)
Other*	8 (1.3)
Prefer not to say	37 (5.8)
Main form of PwD care	
Primary care (e.g., general/family practitioner/internist)	320 (50.0)
Secondary care (e.g., specialist, endocrinologist, diabetologist)	320 (50.0)
Type of specialist	
Primary care	322 (50.3)
Endocrinologists	152 (23.8)
Diabetologists	155 (24.2)
Other	11 (1.7)
Primary work setting	
Physician-owned group practice/private practice	235 (36.7)
Group or community practice	80 (12.5)
Health system-owned practice (academic or non-academic)	76 (11.9)
Public hospital	115 (18.0)
Private hospital	11 (1.7)
University hospital	121 (18.9)
Private university hospital	1 (0.2)
Number of HCPs on diabetes care team in work setting	
Small (1–4)	104 (16.3)
Medium (5–15)	313 (48.9)
Large (> 15)	223 (34.8)
Number of years practicing	
2–5 years	48 (7.5)
> 5 years	592 (92.5)
Years caring for adult PwD with T1D and T2D using insulin pens to treat their diabetes	
2–5 years	53 (8.3)
6–10 years	116 (18.1)
11–20 years	270 (42.2)
21–30 years	165 (25.8)
> 30 years	36 (5.6)
Number of PwD treated with insulin pen (1-week period)	
5–10 PwD	110 (17.2)
> 10 PwD	530 (82.8)

*HCP* healthcare provider, *N* number of HCPs in the study population, *PwD* people with diabetes, *T1D* type 1 diabetes, *T2D* type 2 diabetes

^*^Other ethnic racial background include: no other racial/ethnic backgrounds text description provided

### HCP perceptions of the proportion of PwD with suboptimal dosing and reasons

**Fig. 1:**
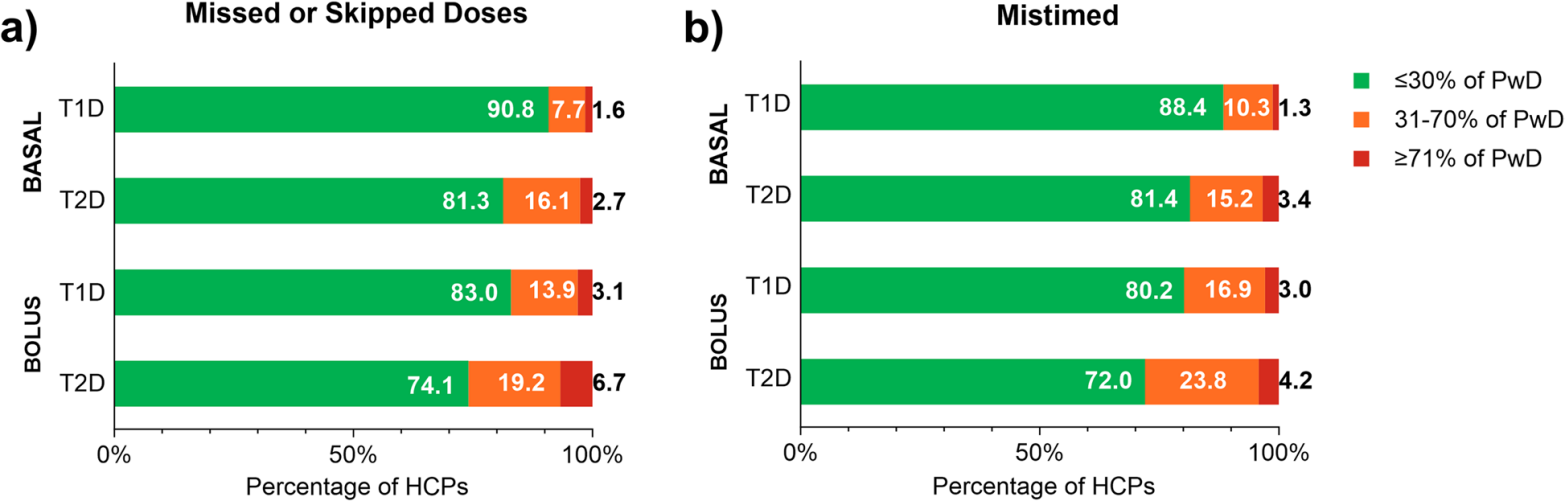
HCP-Estimated Proportion of PwD who a) Missed/Skipped or b) Mistimed Insulin Doses. HCP = healthcare provider; PwD = people with diabetes; T1D = type 1 diabetes; T2D = type 2 diabetes. Note: Percentages may not total 100% due to rounding. The ≤ 30% category comprises the none, 1–10%, 11–20%, and 21–30% subcategories; the 31–70% category comprises the 31–40%, 41–50%, 51–60%, and 61–70% subcategories; and the ≥ 71% category comprises the 71–80%, 81–90%, and 91–100% subcategories. Individual subcategories are presented in Additional File 2, Supplementary Table 1 and Supplementary Table 2. Within the ≤ 30% category, HCP estimated that the proportion of PwD with optimal dosing who did not (**a**) miss or skip any doses constituted 22.2% for basal T1D, 9.2% for basal T2D, 13.6% for bolus T1D, and 6.7% for bolus T2D, while those who did not (**b**) mistime any doses constituted 18.6% for basal T1D, 10.9% for basal T2D, 11.1% for bolus T1D, and 6.9% for bolus T2D. HCP estimates were for their impression of the proportion of PwD with suboptimal dosing in the past 30 days not due to skipping a meal

**Fig. 2:**
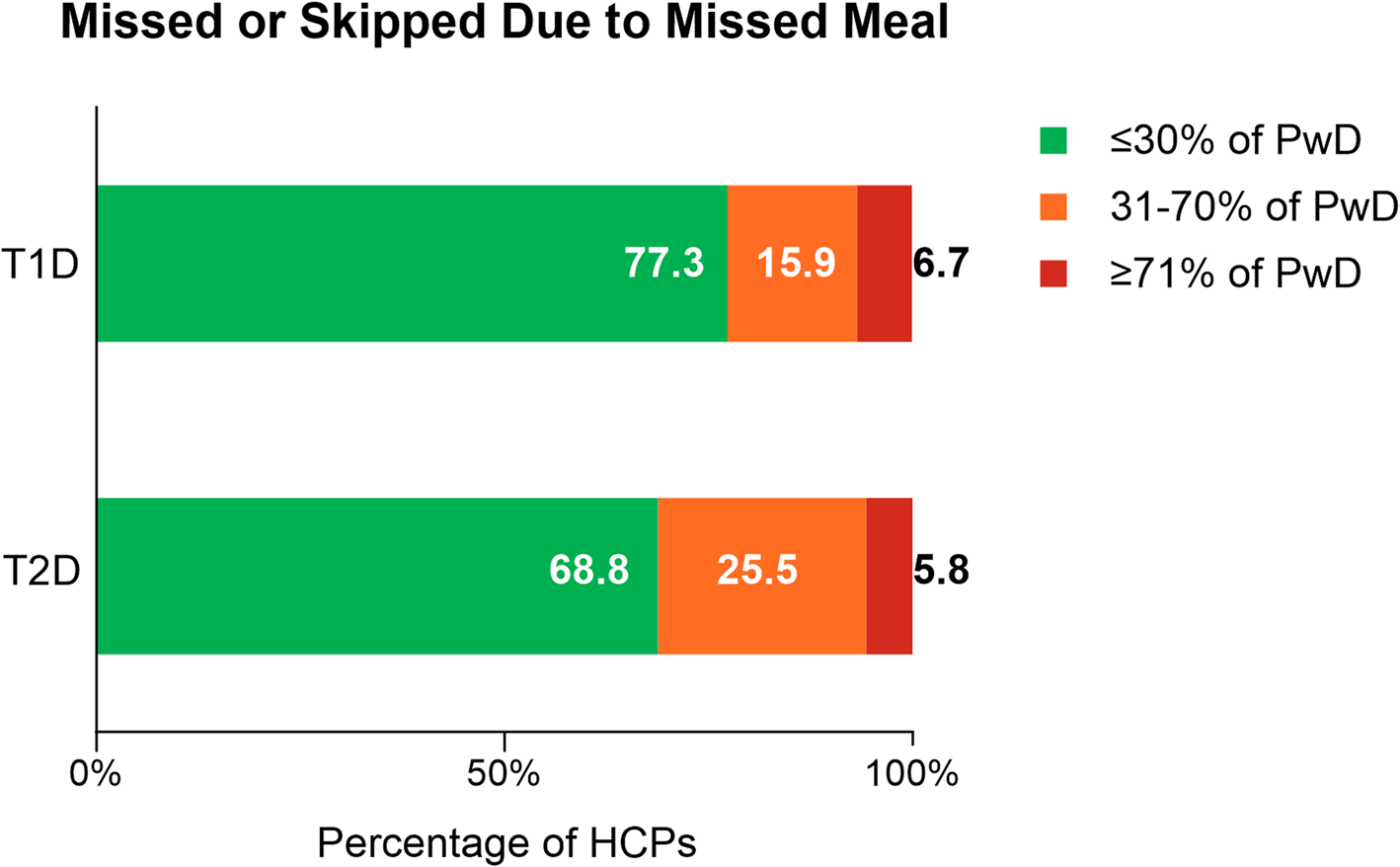
HCP-Estimated Proportion of PwD who Missed/Skipped Their Bolus Insulin Doses. HCP = healthcare provider; PwD = people with diabetes; T1D = type 1 diabetes; T2D = type 2 diabetes. Note: Percentages may not total 100% due to rounding. The ≤ 30% category comprises the none, 1–10%, 11–20%, and 21–30% subcategories; the 31–70% category comprises the 31–40%, 41–50%, 51–60%, and 61–70% subcategories; and the ≥ 71% category comprises the 71–80%, 81–90%, and 91–100% subcategories. Individual subcategories are presented in Additional File 2, Supplementary Table 3. HCP estimates were for their impression of the proportion of PwD with suboptimal dosing in the past 30 days due to skipping a meal. Within the ≤ 30% category, HCPs estimated that 12.5% of those with T1D and 5.2% of those with T2D did not have challenges with missed or skipped doses due to skipped meals

**Fig. 3:**
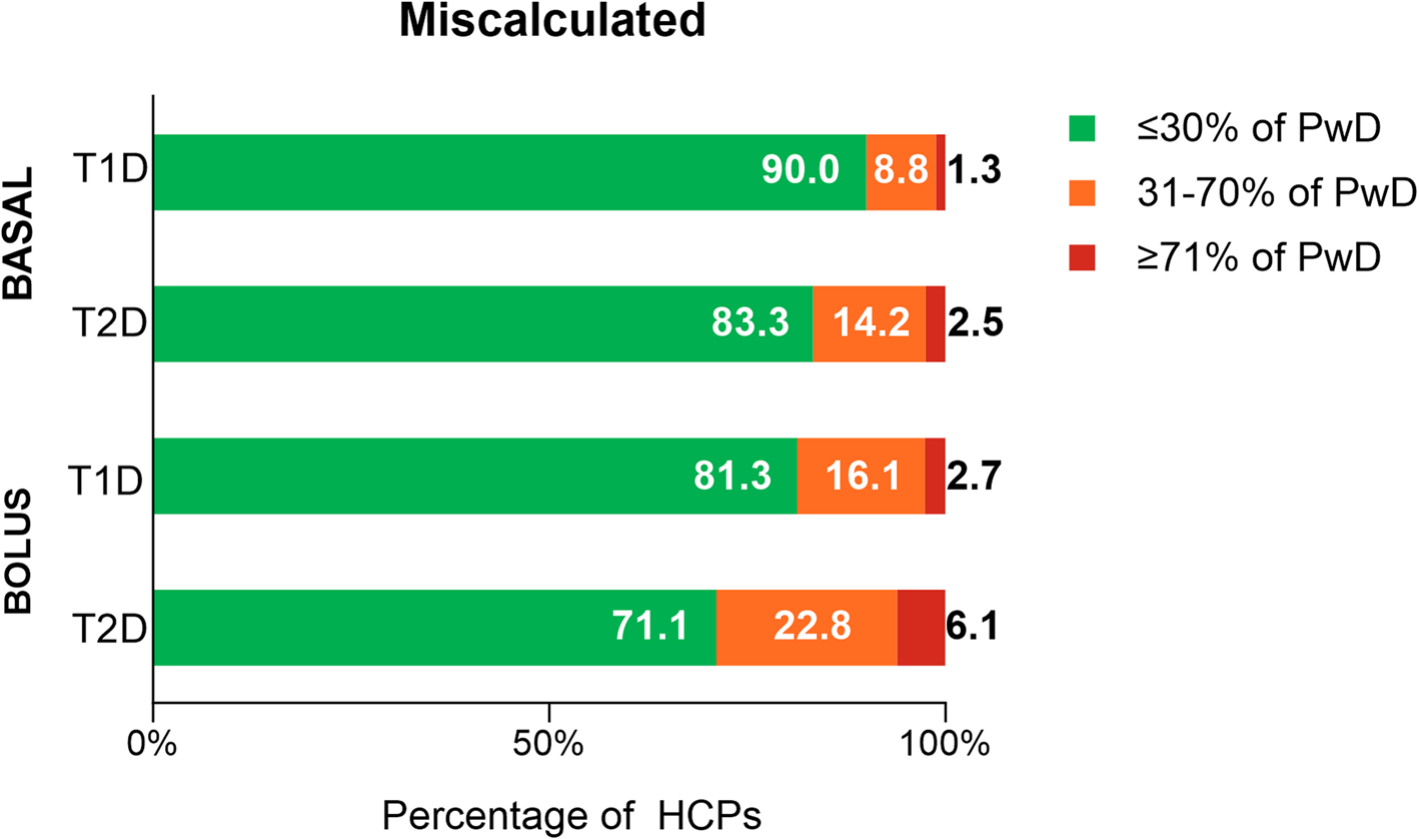
HCP-Estimated Proportion of PwD who Miscalculated Their Insulin Doses. HCP = healthcare provider; PwD = people with diabetes; T1D = type 1 diabetes; T2D = type 2 diabetes. Note: Percentages may not total 100% due to rounding. The ≤ 30% category comprises the none, 1–10%, 11–20%, and 21–30% subcategories; the 31–70% category comprises the 31–40%, 41–50%, 51–60%, and 61–70% subcategories; and the ≥ 71% category comprises the 71–80%, 81–90%, and 91–100% subcategories. Individual subcategories are presented in Additional File 2, Supplementary Table 4. HCP estimates were for their impression of the proportion of PwD with suboptimal dosing in the past 30 days. Within the ≤ 30% category, HCPs estimated that the proportion of PwD who had no challenges in achieving optimal dosing due to calculating doses was 23.9% for basal T1D, 15.0% for basal T2D, 11.3% for bolus T1D, and 5.8% for bolus T2D

HCPs were asked about their perception of the percentage of their patients with T1D or T2D who had missed or skipped bolus and basal insulin doses or who had mistimed these doses in the past 30 days, not due to skipping meals (Fig. 1) (full survey with questions and response options available in Additional File 1). Overall, for T1D and T2D, HCPs most commonly estimated ≤ 30% of PwD missed or skipped a bolus insulin dose without skipping a meal in the last 30 days (bolus dose T1D: 83.0%; T2D: 74.1%) (Fig. 1a). Similar estimates were provided by HCPs for basal insulin dosing (basal dose T1D: 90.8%; T2D: 81.3%). General practitioners and specialists provided similar estimates of the proportions of PwD who they perceived missed or skipped their insulin doses (Table 2). When viewed more closely, HCPs most frequently estimated 1–10% of people with T1D and 1–20% of people with T2D missed or skipped basal and bolus doses not due to missed meals in the past 30 days (Additional File 2, Supplementary Table 1). For mistimed doses, most HCPs estimated ≤ 30% of PwD had mistimed bolus and basal doses without skipping meals (bolus dose T1D: 80.2%; T2D: 72.0%, basal dose T1D: 88.4%; T2D: 81.4%) (Fig. 1b). Within the ≤ 30% category, HCPs most commonly estimated 1–10% of PwD had mistimed doses (Additional File 2, Supplementary Table 2). HCPs were next asked about their perception of the percentage of their patients with T1D or T2D who had missed or skipped bolus insulin doses in the past 30 days, due to instances of skipping meals. The majority of HCPs again estimated ≤ 30% of their PwD had missed bolus doses due to skipping meals (bolus dose T1D: 77.3%; T2D: 68.8%) (Fig. 2 and Additional File 2, Supplementary Table 3). Results for HCP perceptions of the proportion of PwD who miscalculated their basal or bolus insulin dose in the past 30 days are presented in Fig. 3 and Additional File 2, Supplementary Table 4; HCP perceptions of reasons are presented in Table 3.

**Table 2:** General practitioners and specialists estimated proportion of pwd who missed/skipped insulin doses

HCP Estimates of PwD Dosing Behavior	Total Number of HCPs (*N* = 640)			
	General Practitioners (*N* = 320)		Specialists (*N* = 320)	
*n* (%)	Bolus	Basal	Bolus	Basal
Type 1 diabetes				
None	60 (18.8)	87 (27.2)	27 (8.4)	55 (17.2)
≤ 30% of PwD	227 (86.6)	297 (92.8)	224 (79.4)	284 (88.8)
31–70%	39 (12.2)	19 (5.9)	50 (15.6)	30 (9.4)
≥ 71%	4 (1.3)	4 (1.3)	16 (5.0)	6 (1.9)
Type 2 diabetes				
None	35 (10.9)	39 (12.2)	8 (2.5)	20 (6.3)
≤ 30% of PwD	247 (77.2)	266 (83.1)	227 (70.9)	254 (79.4)
31–70%	59 (18.4)	45 (14.1)	64 (20.0)	58 (18.1)
≥ 71%	14 (4.4)	9 (2.8)	29 (9.1)	8 (2.5)

*HCP* healthcare provider, *n* = number of HCPs in the specified category, *N* = number of HCPs in analysis, *PwD* people with diabetes

*Note:* The ≤ 30% category comprises the none, 1–10%, 11–20%, and 21–30% subcategories; the 31–70% category comprises the 31–40%, 41–50%, 51–60%, and 61–70% subcategories; and the ≥ 71% category comprises the 71–80%, 81–90%, and 91–100% subcategories. Individual subcategories are presented in Additional File 2, Supplementary Table 1

HCP estimates were for their impressions of PwD with suboptimal dosing in the past 30 days not due to skipping a meal

**Table 3:** HCP Perceptions of reasons PwD miscalculated basal and bolus insulin doses

Main Reasons for Miscalculated Basal Insulin Dose* n (%)	Total (*N* = 640)	
	Bolus	Basal
Found it too complicated/burdensome	286 (44.7)	189 (29.5)
They were out of their normal routine	305 (47.7)	309 (48.3)
Did not want to dose in front of others	92 (14.4)	109 (17.0)
Not sure how much insulin to take	387 (60.5)	305 (47.7)
Did not measure their blood glucose	349 (54.5)	252 (39.4)
Wanted to avoid their blood sugar getting too low	292 (45.6)	263 (41.1)
Couldn’t remember when last took a dose	146 (22.8)	150 (23.4)
They were trying to save on the cost of insulin	38 (5.9)	28 (4.4)
Other*	3 (0.5%)	6 (0.9)

*HCP* healthcare provider, *n* = number of HCPs in the specified category, *N* = number of HCPs in the study population, *PwD* people with diabetes

^*^Other reasons included: I didn’t have enough insulin with me, I fell asleep, I had forgotten my Lantus at home, I had to check if I had already taken it and when the last dose was, I was too tired and wanted to sleep, I was on a plane and flew through several time zones, over slept, tired, falling asleep before dosing, fasting a.m., in the hospital, out of food, ran out, sick, and unexpected hospital visit due to accident

**Fig. 4:**
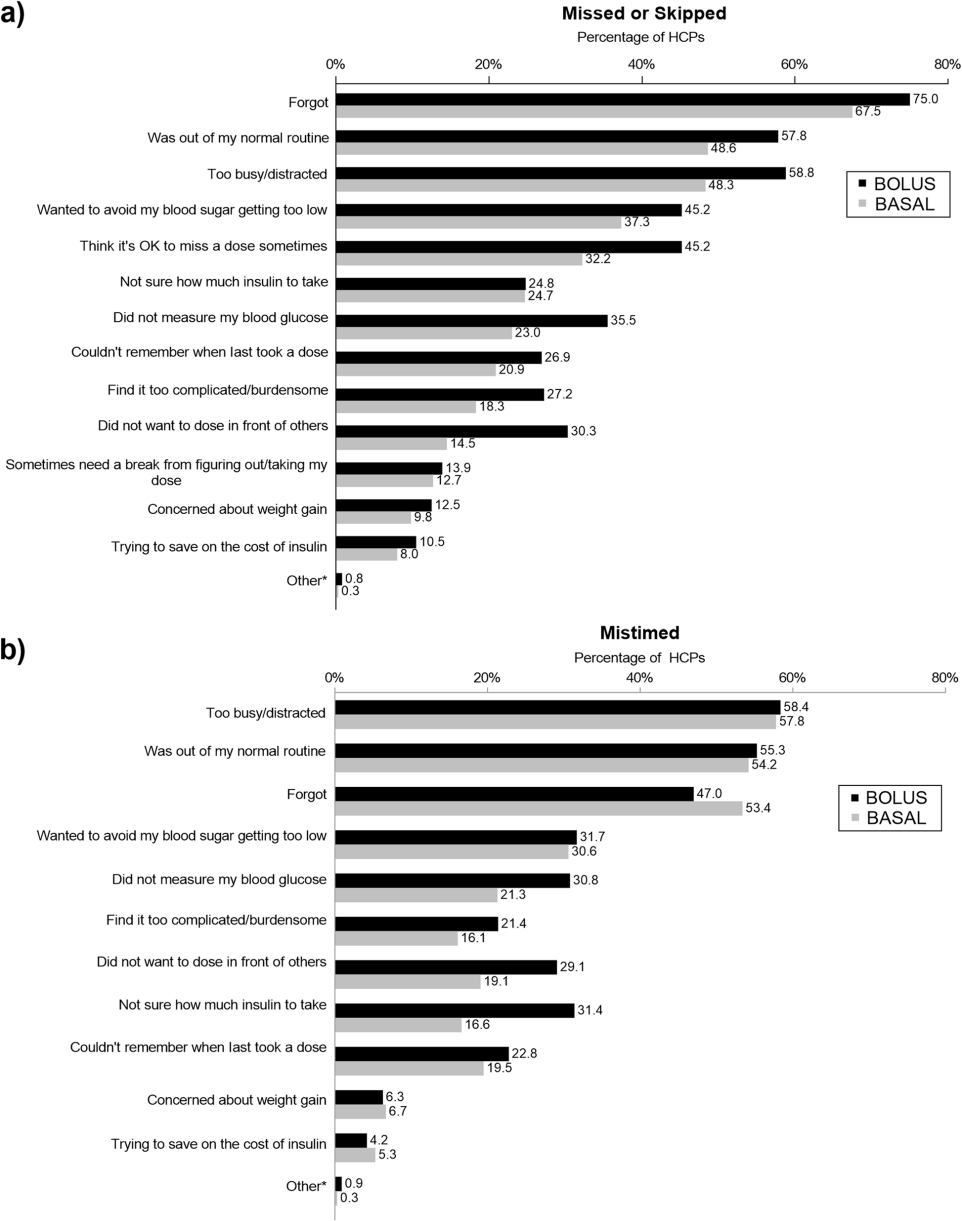
HCP Perceptions of Reasons for **a** Missed/Skipped Insulin Doses and **b** Mistimed Insulin Doses. HCP = healthcare provider. *Other main factors included: they couldn’t remember the plan or were using an old prescription, unsure of the carbohydrate content of the meal, low motive, and poor compliance. Note: HCP estimates for missed/skipped insulin doses were for their impression of the proportion of PwD with suboptimal dosing in the past 30 days, not due to skipping a meal. HCPs were asked to focus on their adult patients withtype 1 and type 2 diabetes who use insulin pens. HCPs were able to select their impression of the 3 main reasons for suboptimal dosing in order of importance

The 3 most common reasons (other than skipping a meal) HCPs endorsed regarding why the PwD missed or skipped insulin doses included they “forgot” (bolus: 75.0%; basal: 67.5%), “were too busy/distracted” (bolus: 58.8%; basal: 48.3%), and “were out of their normal routine” (bolus: 57.8%; basal: 48.6%) (Fig. 4a) (full survey with questions and response options is available in Additional File 1). HCPs reported similar reasons they thought PwD mistimed insulin doses, including they “forgot” (bolus: 47.0%; basal: 53.4%), “were too busy/distracted” (bolus: 58.4%; basal: 57.8%), and “were out of their normal routine” (bolus: 55.3%; basal: 54.2%) (Fig. 4b).

### HCP perceptions of issues/difficulties with aspects of insulin dosing in PwD

A total of 67.5% and 53.6% of HCPs estimated ≤ 30% of PwD with T1D and T2D, respectively, found insulin dosing/management complicated and/or burdensome (Table 4 and Additional File 2, Supplementary Table 5). A total of 55.9% and 53.0% of HCPs reported they were “very confident” in initiating and titrating insulin, respectively, with PwD (Table 5).

**Table 4:** HCP-Estimated proportion of PwD who found Insulin dosing/management complicated and/or burdensome

	**PwD Categories**	**Total** **(N = 640)**
**T1D**	None	25 (3.9)
	≤ 30%	432 (67.5)
	31–70%	145 (22.7)
	≥ 71%	42 (6.6)
	Missing*	21 (3.3)
**T2D**	None	15 (2.3)
	≤ 30%	343 (53.6)
	31–70%	231 (36.1)
	≥ 71%	62 (9.7)

*HCP* healthcare provider, *N* = number of HCPs in the study population, *PwD* people with diabetes, *T1D* type 1 diabetes, *T2D* type 2 diabetes

*Note:* The ≤ 30% category comprises the none, 1–10%, 11–20%, and 21–30% subcategories; the 31–70% category comprises the 31–40%, 41–50%, 51–60%, and 61–70% subcategories; and the ≥ 71% category comprises the 71–80%, 81–90%, and 91–100% subcategories. Individual subcategories are presented in Additional File 2, Supplementary Table 5

^*^Missing counts are participants who answered “none” to Survey Question 6, which asked “approximately how many adult patients on insulin pens are you currently treating?”

**Table 5:** HCP Confidence in initiating and titrating insulin with PwD

	HCP Confidence	Total (*N* = 640)
Initiating insulin	Very confident	358 (55.9)
	Somewhat confident	220 (34.4)
	Neutral	60 (9.4)
	Not confident	2 (0.3)
Titrating insulin	Very confident	339 (53.0)
	Somewhat confident	241 (37.7)
	Neutral	53 (8.3)
	Not confident	7 (1.1)

*HCP* healthcare provider, *N* = number of HCPs in the study population, *PwD* people with diabetes

**Fig. 5:**
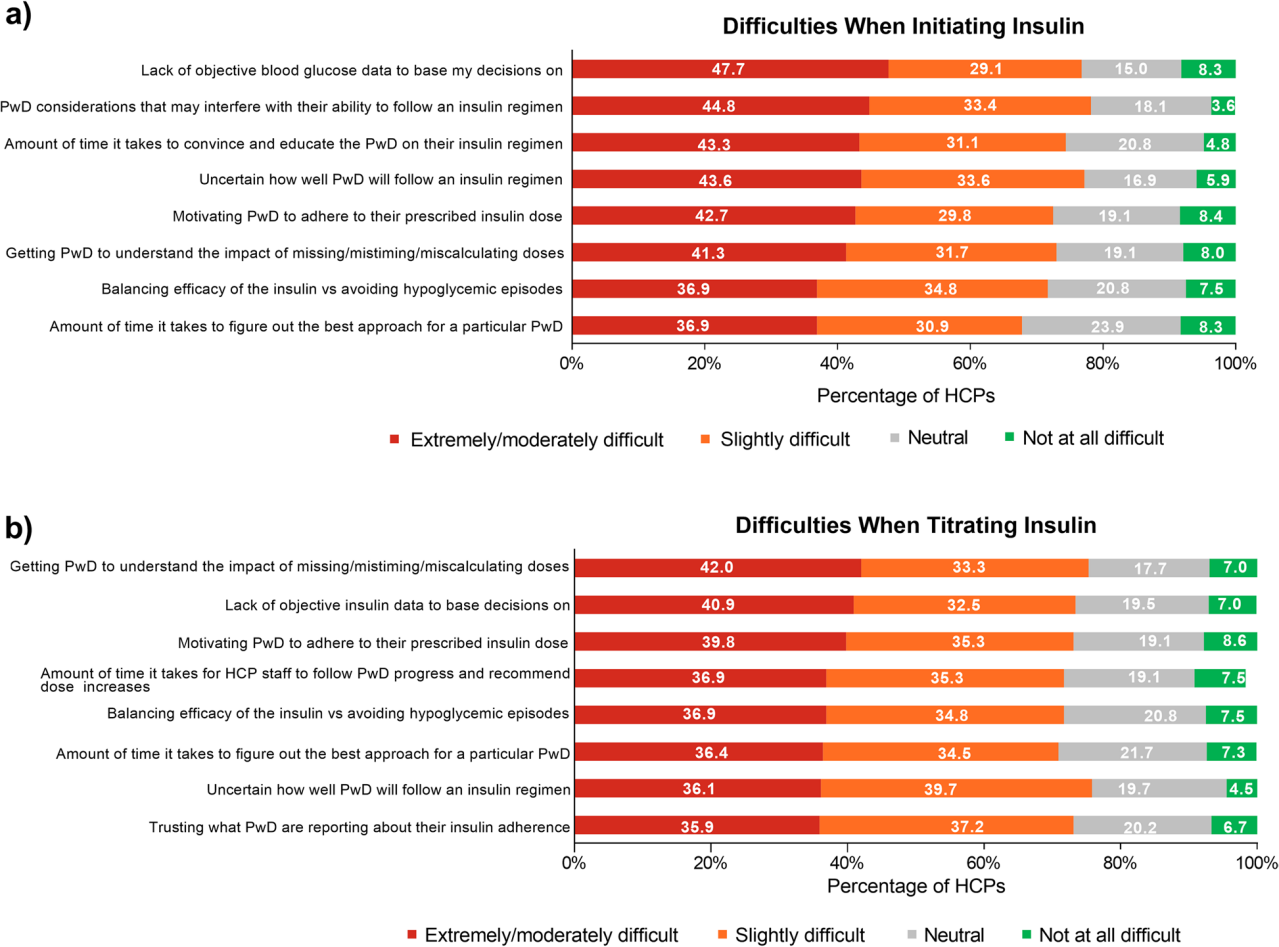
HCP Perceptions of Difficulty in **a** Initiating Insulin and **b** Titrating Insulin. HCP = healthcare provider. HCPs were asked to focus on their adult patients withtype 1 and type 2 diabetes who use insulin pens

Overall, HCPs found it “extremely or moderately difficult” to motivate PwD to self-manage their agreed-upon insulin dose when initiating insulin (*n* = 273/640, 42.7% of HCPs) or titrating insulin (*n* = 255/640, 39.8% of HCPs) (Fig. 5). Other aspects of initiating insulin that HCPs found “extremely or moderately difficult” included “lack of objective blood glucose data to base my decisions on” (*n* = 305/640, 47.7%), “PwD considerations that may interfere with their ability to follow an insulin regimen” (*n* = 287/640, 44.8%), and “uncertain how well PwD will follow an insulin regimen” (*n* = 279/640, 43.6%) (Fig. 5a). With respect to titrating insulin, HCPs found the following “extremely or moderately difficult”: “lack of objective blood glucose data to base my decisions on” (*n* = 280/640, 43.8%) and “getting PwD to understand the impact of missing/mistiming/miscalculating doses” (*n* = 269/640 42.0%) (Fig. 5b). For T1D, with respect to bolus and basal dosing routines, 39.1% and 57.0% of HCPs, respectively, felt 71–100% of PwD fully self-managed their prescribed dose; for T2D, with respect to bolus and basal dosing routines, 21.7% and 40.6% of HCPs, respectively, felt 71–100% of PwD fully self-managed their prescribed dose (Table 6 and Additional File 2, Supplementary Table 6).

**Table 6:** HCP-Estimated proportion of PwD who do not struggle with their prescribed insulin dosing routine

HCP Estimates of PwD Adherence	Total Number of HCPs (*N* = 640)	
*n*(%)	Bolus	Basal
Type 1 Diabetes		
None	3 (0.5)	5 (0.8)
≤ 30% of PwD	158 (24.7)	107 (16.7)
31–70%	211 (33.0)	147 (23.0)
≥ 71%	250 (39.1)	365 (57.0)
Missing*	21 (3.3)	21 (3.3)
Type 2 Diabetes		
None	6 (0.9)	8 (1.3)
≤ 30% of PwD	188 (29.4)	119 (18.6)
31–70%	309 (48.3)	257 (40.2)
≥ 71%	139 (21.7)	260 (40.6)
Missing*	4 (0.6)	4 (0.6)

*HCP* healthcare provider, *n* = number of HCPs in the specified category, *N* = number of HCPs in analysis population, *PwD* people with diabetes

*Note:* The ≤ 30% category comprises the none, 1–10%, 11–20%, and 21–30% subcategories; the 31–70% category comprises the 31–40%, 41–50%, 51–60%, and 61–70% subcategories; and the ≥ 71% category comprises the 71–80%, 81–90%, and 91–100% subcategories. Individual subcategories are presented in Additional File 2, Supplementary Table 6

^*^ Missing counts are participants who answered “none” to Survey Question 6, which asked “approximately how many adult patients on insulin pens are you currently treating?”

### Solutions suggested by HCPs for optimizing insulin dosing in PwD

**Fig. 6:**
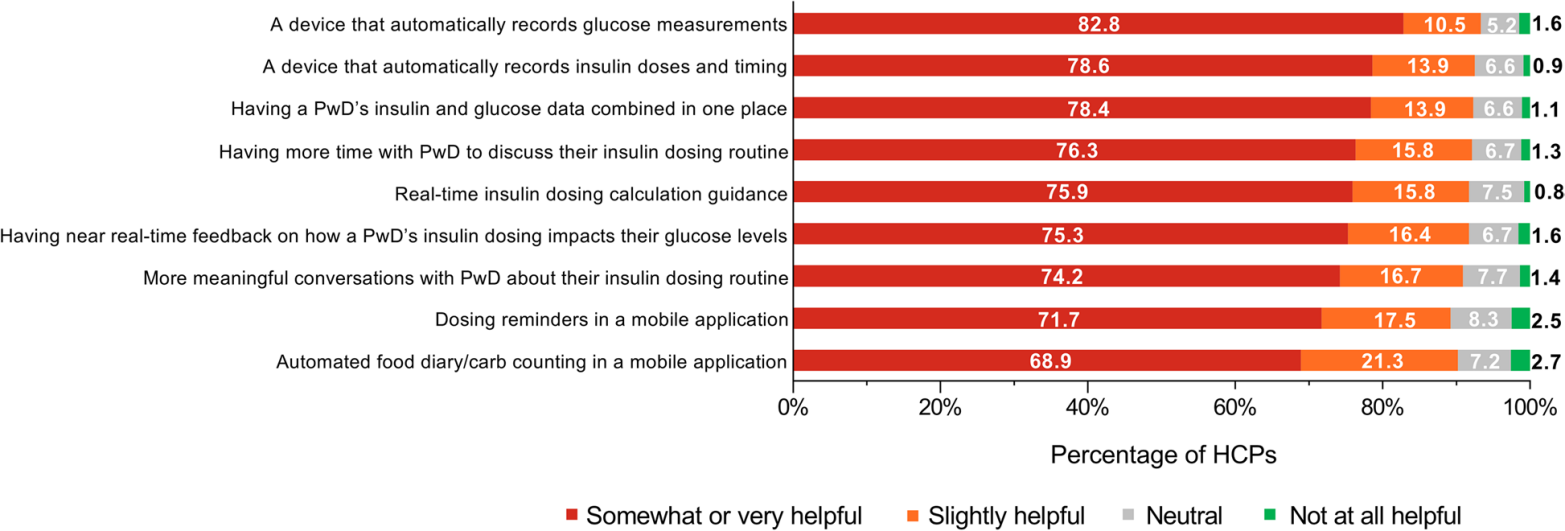
HCP Opinions on Solutions That Could Help PwD Optimize Their Insulin Dosing. HCP = healthcare provider; PwD = people with diabetes. HCPs were asked to focus on their adult patients with type 1 and type 2 diabetes who use insulin pens

With respect to optimizing insulin dosing, > 75% of HCPs considered it somewhat or very helpful for PwD to have (Fig. 6):

- A device that automatically records glucose measurements
- A device that automatically records insulin doses and timing
- Insulin and glucose data combined in one place
- More time to discuss their insulin dosing routine
- Real-time insulin dosing calculation guidance
- Near real-time feedback on how insulin dosing impacts their glucose levels

## Discussion

This study aimed to examine the extent of suboptimal insulin dosing and current barriers to optimal insulin dosing in PwD, as seen from the HCP perspective. The majority of HCPs estimated ≤ 30% of patients with T1D and/or T2D missed or skipped bolus or basal insulin doses (not due to missing a meal). The top reasons HCPs estimated for missing these doses were PwD forgetting, being busy or distracted, or being out of their normal routine. A majority of HCPs also estimated ≤ 30% of PwD with T1D or T2D found insulin management complicated or burdensome. A total of 55.9% and 53.0% of HCPs reported being very confident in initiating insulin and titrating insulin with PwD, respectively, and 42.7% and 39.8% of HCPs found motivating PwD to self-manage the agreed-upon insulin dose “extremely or moderately difficult” when initiating insulin or titrating insulin, respectively.

There may be a disconnect between HCP perception and PwD-reported dosing behaviors. The current study found most HCPs estimated fewer than 30% of their patients had missed or skipped insulin doses in the past 30 days. HCPs most frequently estimated 1–10% of people with T1D missed or skipped doses, and 1–20% of people with T2D missed or skipped insulin doses in the past 30 days. In a study of PwD (*N* = 1530), 35% of participants reported challenges in following agreed-upon insulin treatment plans 1 or more days per month [8]. In another study of PwD (*N* = 1150), between 48% (basal) and 56% (bolus) of participants reported missing doses, while 40% (bolus) to 46% (basal) of participants reported mistimed doses in the past 30 days [7]. Although direct comparison is not possible, these apparent discrepancies suggest communication between PwD and HCPs varies from the information reported by patients about their dosing and self-management of treatment. HCPs may be under-informed and thus underestimate the extent of suboptimal dosing of the PwD they are treating.

HCPs found it “extremely or moderately difficult” to motivate PwD to self-manage their agreed-upon dose, whether initiating insulin or titrating insulin. Additionally, one of the primary aspects of initiating or titrating insulin doses HCPs found “extremely or moderately difficult” included “lack of objective blood glucose data to base my decisions on.” These results highlight what HCPs find challenging about helping patients manage their treatment and indicate additional training may be needed to help HCPs support PwD more successfully in optimal insulin management.

In addition to identifying reasons HCPs believe PwD exhibit suboptimal insulin dosing and experience challenges in dosing management, the survey reported potential solutions for optimizing insulin dosing from the HCP perspective. The top 3 solutions that HCPs suggested could help PwD optimize their insulin dosing included “a device that automatically records glucose measurements,” “a device that automatically records insulin doses and timing,” and “having the insulin and glucose data of a PwD combined in one place.” These aspects of digital technology, including data visibility and automation, may help ensure HCPs and PwD both have the same objective information about missed and mistimed doses in a timely manner [1]. The increased adoption of technologies designed to assist with self-management, such as continuous glucose monitors, connected insulin pens, and automated insulin delivery systems, may alleviate or reduce some of these challenges when coupled with education, follow-up, and support [9]. Automated real-time data could enable HCPs and patients to have more constructive conversations about prescribed treatments. Additionally, receiving data in real-time could help fine-tune a personalized dosing regimen and aid in self-management of insulin dosing.

The fourth most common solution suggested by HCPs to help PwD optimize their insulin dosing was “having more time with my patient to discuss their insulin dosing routines.” HCPs are aware that increased communication with PwD will aid in better insulin dosing and achieving glycemic goals. Given this awareness and the differences between PwD and HCP survey responses regarding suboptimal insulin dosing, developing strategies for better communication between PwD and their HCPs is an urgent need.

Overall, the results of this survey, in the context of other published findings, suggest there is a disconnect between HCP perceptions and the self-reported dosing behaviors of PwD. Furthermore, HCPs perceive suboptimal insulin dosing in PwD occurs for a variety of intentional and unintentional reasons. Approaches that can help improve the objectivity of dose measurements for both PwD and HCPs and increase associated communications may reduce suboptimal insulin dosing and improve treatment outcomes.

A notable strength of this survey study is the large population size of HCPs (*N* = 640), which increases the generalizability of the survey results. The survey also ensured good representation of both specialist and primary care work settings. With awareness of potential bias of self-reported survey data such as recall error and exclusions of nuanced information by the use of closed-ended survey questions, a pilot study was conducted to assess the questionnaire under survey conditions to first examine its validity. Revision of the survey questions after pilot study feedback subsequently aimed to limit bias and ensure all desired information was captured. Statistical comparison between the survey of HCP perceived compliance of PwD reported here and a survey of PwD self-reported compliance which was conducted in parallel [7] was not planned, as the surveys were designed as 2 independent samples. HCPs can help PwD to achieve optimal dosing if they have better objective insights into actual dosing levels, which could improve glucose outcomes. Due to the non-interventional study design, there was no direct assessment of the impact of HCP perspectives on glucose outcomes among PwD. This study did not address difficulties experienced by PwD or challenges to achieving optimal self-management of treatment, which omits a major issue that HCPs must address when facing the challenge of suboptimal insulin dosing. Further research exploring these concepts will help to drive this agenda forward.

## Conclusions

The results of this study indicate that HCPs believe the extent of suboptimal insulin dosing is limited in frequency. While no direct comparisons were made in this study, prior studies in PwD show that self-reported suboptimal insulin dosing frequently occurs. Compared with other studies, the results of this survey suggest a disconnect between HCP perceptions and the actual self-reported dosing behaviors of PwD and that further studies are warranted. Based on perceived barriers and potential solutions from the HCP perspective, approaches that can increase the objectivity of dose measurements and improve communication for both PwD and HCPs may reduce suboptimal insulin dosing and improve treatment outcomes in PwD.

### Supplementary Information

**Supplementary Material 1.**

**Supplementary Material 2.**

## Data Availability

Eli Lilly and Company provides access to all individual participant data collected during the trial, after anonymization, with the exception of pharmacokinetic or genetic data. Data are available to request 6 months after the indication studied has been approved in the US and EU and after primary publication acceptance, whichever is later. No expiration date of data requests is currently set once data are made available. Access is provided after a proposal has been approved by an independent review committee identified for this purpose and after receipt of a signed data sharing agreement. Data and documents, including the study protocol, statistical analysis plan, clinical study report, and blank or annotated case report forms, will be provided in a secure data sharing environment. For details on submitting a request, see the instructions provided at www.vivli.org.

## Abbreviations

HCP: Healthcare provider
PwD: People with diabetes
T1D: Type 1 diabetes
T2D: Type 2 diabetes
