# Supplementary material for: Understanding suboptimal insulin use in type 1 and 2 diabetes: a cross-sectional survey of healthcare providers who treat people with diabetes
Source: BMC Prim Care. 2024 Apr 22;25:124. doi: 10.1186/s12875-024-02390-9 (PMC11034124; doi:10.1186/s12875-024-02390-9)
Supplement: Supplementary file 1 — Supplementary Material 1. [file 12875_2024_2390_MOESM1_ESM.pdf]

2020-9210

Type 1 Diabetes Mellitus (T1DM) and Type 2 Diabetes Mellitus (T2DM) Main Survey 2.0

Healthcare Professional (HCP) Survey

Content

1. ELIGIBILITY SCREENER; 7 questions
2. INTRODUCTION TO SURVEY
3. INFORMED CONSENT FORM (ICF)
4. CURRENT TREATMENT AND ROUTINES; 12 questions
5. INSULIN DOSING ROUTINES OF PATIENTS; 19 questions
6. INSULIN DOSING ISSUES, SATISFACTION, AND EXPECTATIONS; 15 questions

Total questions: 53

Total questions excluding eligibility screener and informed consent form: 46

Thank you for your interest in this study, please answer the following questions so we can assess whether you are eligible to participate.

Please answer all the questions you are asked.

Eligibility Screener

1. **Which of the following diseases or conditions do you diagnose or treat regularly in your practice?**

   Please select all that apply:

   ☐ Asthma

   ☐ Cancer

   ☐ Chronic obstructive pulmonary disease

   ☐ Depression

   ☐ Diabetes mellitus [If not selected – Terminate]

   ☐ High cholesterol

   ☐ Hypertension

   ☐ Migraine

   ☐ Psoriasis

   ☐ Pituitary disease

   ☐ Rheumatoid arthritis

   ☐ Thyroid disease

   ☐ None of the above - [If selected - Terminate]

2. **Do you initiate insulin for adult patients with diabetes?**

   Please select only one:

   ☐ Yes

   ☐ No [If selected - Terminate]

3. **What main form of patient care are you involved in?**

   Please select only one:

   ☐ Primary Care (e.g., general/family practitioner/internist) [Limit to n=80 per country, once target achieved, terminate subsequent entries]

   ☐ Secondary Care (e.g., specialist, endocrinologist, diabetologist) [Limit to n=80 per country, once target achieved, terminate subsequent entries]

4. **How long ago did you graduate as a qualified professional to treat adult patients with diabetes?**

   Please select only one:

   ☐ Less than 2 years [If selected - Terminate]

☐ Between 2 and 5 years

☐ More than 5 years

5. How many adult patients with diabetes on an insulin pen regimen do you see in an average week?

   Please select only one:

   ☐ Less than 5 patients [If selected - Terminate]

   ☐ Between 5 and 10 patients

   ☐ More than 10 patients

6. What is your gender?

   Please check only one:

   ☐ Male

   ☐ Female

   ☐ Trans male/trans man

   ☐ Trans female/trans woman

   ☐ Gender non-conforming/gender queer

   ☐ Prefer not to say

   ☐ Other

7. What is your racial/ethnic background?

   Please check as many as apply:

   ☐ White

   ☐ Black or African American

   ☐ Asian

   ☐ Hispanic or Latino

   ☐ Native Hawaiian or other Pacific Islander

   ☐ American Indian or Alaskan Native

   ☐ Prefer not to say

   ☐ Other (please specify) [Open Text Field]

ELIGIBLE: Those who are eligible will continue to the introduction of the survey and informed consent form.

NOT ELIGIBLE: Those who are not eligible will see the following text: *Thank you for your interest in this research; however, you are not eligible to participate in the current study.*

Introduction to Survey

Title: Type 1 Diabetes Mellitus (T1DM) and Type 2 Diabetes Mellitus (T2DM) Healthcare Professional (HCP) Survey

Thank you for agreeing to participate in this survey regarding perceptions of patients with diabetes. In this questionnaire we will ask about your views on insulin usage and dosing and patient adherence. When answering these questions please focus on your ***adult patients with type 1 and type 2 diabetes mellitus that use insulin pens***. Please try to answer each question to the best of your ability. The survey will take about 45 minutes to complete. After you complete the survey, you will be remunerated as outlined by your panel.

Informed Consent Form (ICF)

As per protocol

Current Treatment and Routines

We would like to know about your experience treating patients with diabetes.

When answering the questions in this section, please refer only to adult patients with T1DM and T2DM using insulin pens – excluding those on pre-mixed pens.

1. How many years have you been qualified to practice medicine?

   Please select only one

   ☐ 2-5 years

   ☐ 6-10 years

   ☐ 11-20 years

   ☐ 21-30 years

   ☐ 30-39 years

   ☐ More than 40 years

2. How many years have you been caring for adult patients with T1DM and T2DM using insulin pens to treat their diabetes?

   Please select only one

   ☐ 2-5 years

   ☐ 6-10 years

   ☐ 11-20 years

   ☐ 21-30 years

   ☐ 30-39 years

   ☐ More than 40 years

3. What form of medicine do you practice?

   Please select only one

   ☐ Family practitioner/internist/general practitioner/primary care

   ☐ Endocrinologist

   ☐ Diabetologist

   ☐ Other (please specify) [Open Text Field]

4. What type of setting do you <u>mostly</u> work in?

   Please select only one

   ☐ Physician-owned group practice/private practice

☐ Group or community practice

☐ Health system-owned practice (academic or non-academic)

☐ Public hospital

☐ Private hospital

☐ University hospital

☐ Private University hospital

☐ Locum

☐ Other

5. **How many healthcare personnel are part of the diabetes care team in your practice setting (note: healthcare personnel includes medical assistants, dietitians, nurses, general practitioners, and specialists)?**

   Please select only one

   ☐ Small (1–4 healthcare professionals)

   ☐ Medium (5–15 healthcare professionals)

   ☐ Large (more than 15 healthcare professionals)

6. **Approximately how many adult patients on insulin pens are you currently treating?**

   Please select only one for each column

   ☐ Type 1 diabetes

   - ☐ None
   - ☐ 1-10 patients
   - ☐ 11-20 patients
   - ☐ 21-30 patients
   - ☐ 31-50 patients
   - ☐ 51-70 patients
   - ☐ 71-90 patients
   - ☐ 91-120 patients
   - ☐ 121-150 patients
   - ☐ More than 150 patients

   ☐ Type 2 diabetes

   - ☐ None

☐ 1-10 patients

☐ 11-20 patients

☐ 21-30 patients

☐ 31-50 patients

☐ 51-70 patients

☐ 71-90 patients

☐ 91-120 patients

☐ 121-150 patients

☐ More than 150 patients

7. Which healthcare professionals are part of the treating team where you manage your adult patients using insulin pens?

   Please select all that apply

   For type 1 patients (Include if > 0 type 1 diabetes patients were selected in Q6)

   ☐ Dietician

   ☐ Nurse or diabetes nurse educator

   ☐ Physical therapist

   ☐ General practitioner

   ☐ Endocrinologist

   ☐ Diabetologist

   ☐ Other (please specify) [Open Text Field]

   For type 2 patients (Include if > 0 type 2 diabetes patients were selected in Q6)

   ☐ Dietician

   ☐ Nurse or diabetes nurse educator

   ☐ Physical therapist

   ☐ General practitioner

   ☐ Endocrinologist

   ☐ Diabetologist

   ☐ Other (please specify) [Open Text Field]

8. On average, how often do you see adult patients treated with insulin pens in your clinic for diabetes management?

Patients with type 1 diabetes (Include if > 0 type 1 diabetes patients were selected in Q6)

Please select only one

☐ Once a month or more

☐ Once every 2–3 months

☐ Once every 4–6 months

☐ Every 6 months or less

Patients with type 2 diabetes (Include if > 0 type 2 diabetes patients were selected in Q6)

Please select only one

☐ Once a month or more

☐ Once every 2–3 months

☐ Once every 4–6 months

☐ Every 6 months or less

9. Overall, what proportion of your type 2 diabetes mellitus patients using insulin pens follow a BASAL + BOLUS regime? (Include if > 0 type 2 diabetes patients were selected in Q6)

Please select only one

☐ None

☐ 1-10% of patients

☐ 11-20% of patients

☐ 21-30% of patients

☐ 31-40% of patients

☐ 41-50% of patients

☐ 51-60% of patients

☐ 61-70% of patients

☐ 71-80% of patients

☐ 81-90% of patients

☐ 91-100% of patients

10. Overall, what proportion of your type 2 diabetes mellitus patients on insulin pens follow a BASAL ONLY regime? (Include if > 0 type 2 diabetes patients were selected in Q6)

Please select only one

☐ None

☐ 1-10% of patients

☐ 11-20% of patients

☐ 21-30% of patients

☐ 31-40% of patients

☐ 41-50% of patients

☐ 51-60% of patients

☐ 61-70% of patients

☐ 71-80% of patients

☐ 81-90% of patients

☐ 91-100% of patients

11. In the last 12 months, what proportion of your <u>type 1</u> diabetes mellitus patients have experienced <u>severe</u> low blood sugar (hypoglycemic) events?

Please select only one

☐ None

☐ 1-10% of patients

☐ 11-20% of patients

☐ 21-30% of patients

☐ 31-40% of patients

☐ 41-50% of patients

☐ 51-60% of patients

☐ 61-70% of patients

☐ 71-80% of patients

☐ 81-90% of patients

☐ 91-100% of patients

12. In the last 12 months, what proportion of your <u>type 2</u> diabetes mellitus patients have experienced <u>severe</u> low blood sugar (hypoglycemic) events?

Please select only one

☐ None

☐ 1-10% of patients

☐ 11-20% of patients

☐ 21-30% of patients

☐ 31-40% of patients

☐ 41-50% of patients

☐ 51-60% of patients

☐ 61-70% of patients

☐ 71-80% of patients

☐ 81-90% of patients

☐ 91-100% of patients

Insulin Dosing Routines of Patients

MISSED INSULIN DOSES

The following questions ask you about any insulin doses that you think your patients may have missed or skipped. By missing a dose, we mean any time they have not taken a dose that they should have taken, intentionally (on purpose) or unintentionally (not on purpose or forgotten).

When answering the questions in this section, please refer only to adult patients with T1DM and T2DM using insulin pens.

1. **In the past 30 days, what proportion of your patients with type 1 or 2 diabetes mellitus do you think missed, or skipped, BOLUS insulin doses due to skipping a meal?**

   Please select only one for each column

   ☐ Of all patients with type 1 diabetes mellitus who use BOLUS insulin

   - ☐ None
   - ☐ 1-10% of patients
   - ☐ 11-20% of patients
   - ☐ 21-30% of patients
   - ☐ 31-40% of patients
   - ☐ 41-50% of patients
   - ☐ 51-60% of patients
   - ☐ 61-70% of patients
   - ☐ 71-80% of patients
   - ☐ 81-90% of patients
   - ☐ 91-100% of patients

   ☐ Of all patients with type 2 diabetes mellitus who use BOLUS insulin

   - ☐ None
   - ☐ 1-10% of patients
   - ☐ 11-20% of patients
   - ☐ 21-30% of patients
   - ☐ 31-40% of patients
   - ☐ 41-50% of patients
   - ☐ 51-60% of patients
   - ☐ 61-70% of patients

☐ 71-80% of patients

☐ 81-90% of patients

☐ 91-100% of patients

2. **In the past 30 days, what proportion of your patients with type 1 or 2 diabetes mellitus do you think missed, forgot, or skipped, BOLUS insulin doses (not due to skipping a meal)?**

   Please select only one for each column

   ☐ Of all patients with type 1 diabetes mellitus who use BOLUS insulin

   - ☐ None
   - ☐ 1-10% of patients
   - ☐ 11-20% of patients
   - ☐ 21-30% of patients
   - ☐ 31-40% of patients
   - ☐ 41-50% of patients
   - ☐ 51-60% of patients
   - ☐ 61-70% of patients
   - ☐ 71-80% of patients
   - ☐ 81-90% of patients
   - ☐ 91-100% of patients

   ☐ Of all patients with type 2 diabetes mellitus who use BOLUS insulin

   - ☐ None
   - ☐ 1-10% of patients
   - ☐ 11-20% of patients
   - ☐ 21-30% of patients
   - ☐ 31-40% of patients
   - ☐ 41-50% of patients
   - ☐ 51-60% of patients
   - ☐ 61-70% of patients
   - ☐ 71-80% of patients
   - ☐ 81-90% of patients
   - ☐ 91-100% of patients

3. In the past 30 days, what do you believe were the main factors (other than skipping a meal) that caused your patients with diabetes type 1 or 2 to miss, or skip, a BOLUS insulin dose? (Only show for respondents who have indicated that their patients miss doses)

   Please select all that apply:

   ☐ They forgot

   ☐ They were too busy/distracted

   ☐ They sometimes need a break from figuring out/taking their dose

   ☐ They think it's ok to miss a dose sometimes

   ☐ They find it too complicated and burdensome

   ☐ They were out of their normal routine

   ☐ They did not want to dose in front of others

   ☐ They weren't sure how much insulin to take so didn't take any (e.g., sick, exercising, unfamiliar food, didn't have my insulin plan available)

   ☐ They couldn't remember when they last took a dose

   ☐ They did not measure their blood glucose

   ☐ They wanted to avoid their blood sugar getting too low

   ☐ They were concerned about weight gain

   ☐ They wanted to save on the cost of insulin

   ☐ Other (please specify) [Open Text Field]

4. Please rank in order from 1-3 the most common reasons from those you have selected as the reasons why your patients missed, or skipped, a BOLUS insulin dose.

   Please select your top three most common reasons and rank them in order, using the drag and drop function below.

   [Ranking exercise from 1 to 3 repeating all responses selected in Q3]

5. In the past 30 days, what proportion of your patients with type 1 or 2 diabetes mellitus do you think missed, or skipped, BASAL insulin doses (not due to skipping a meal)?

   ☐ Of all patients with <u>type 1</u> diabetes mellitus

      Please select one for each column

      ☐ None

      ☐ 1-10% of patients

      ☐ 11-20% of patients

      ☐ 21-30% of patients

☐ 31-40% of patients

☐ 41-50% of patients

☐ 51-60% of patients

☐ 61-70% of patients

☐ 71-80% of patients

☐ 81-90% of patients

☐ 91-100% of patients

☐ Of all patients with type 22 diabetes mellitus

☐ None

☐ 1-10% of patients

☐ 11-20% of patients

☐ 21-30% of patients

☐ 31-40% of patients

☐ 41-50% of patients

☐ 51-60% of patients

☐ 61-70% of patients

☐ 71-80% of patients

☐ 81-90% of patients

☐ 91-100% of patients

6. In the past 30 days, what do you believe were the main factors (other than skipping a meal) that caused your patients with diabetes type 1 or 2 to miss, or skip, a BASAL insulin dose? (Only show for respondents who have indicated that their patients miss doses)

Please select all that apply:

☐ They forgot

☐ They were too busy/distracted

☐ They sometimes need a break from figuring out/taking their dose

☐ They think it's ok to miss a dose sometimes

☐ They find it too complicated and burdensome

☐ They were out of their normal routine

☐ They did not want to dose in front of others

☐ They weren't sure how much insulin to take so I didn't take any (e.g., sick, exercising, unfamiliar food, didn't have my insulin plan available)

☐ They couldn't remember when they last took a dose

☐ They did not measure their blood glucose

☐ They wanted to avoid their blood sugar getting too low

☐ They were concerned about weight gain

☐ They wanted to save on the cost of insulin

☐ Other (please specify) [Open Text Field]

7. **Please rank in order from 1-3 the most common reasons from those you have selected as the reasons why your patients missed, or skipped, a BASAL insulin dose.**

   Please select your top three most common reasons and rank them in order, using the drag and drop function below.

   [Ranking exercise from 1 to 3 repeating all responses selected in Q6]

<u>MISTIMED INSULIN DOSES</u>

The following questions ask you about any insulin doses that your patients may have taken at the wrong time (e.g., not within 10 to 15 minutes before a meal BOLUS/mealtime insulin, or not at the usual time for BASAL insulin).

When answering the questions in this section, please refer only to adult patients with T1DM and T2DM using insulin pens.

8. **In the past 30 days, what proportion of your patients with type 1 or 2 diabetes mellitus do you think mistimed BOLUS insulin doses?**

   Please select only one for each column

   ☐ Of all patients with type 1 diabetes mellitus who use BOLUS insulin

   - ☐ None
   - ☐ 1-10% of patients
   - ☐ 11-20% of patients
   - ☐ 21-30% of patients
   - ☐ 31-40% of patients
   - ☐ 41-50% of patients
   - ☐ 51-60% of patients
   - ☐ 61-70% of patients
   - ☐ 71-80% of patients

☐ 81-90% of patients

☐ 91-100% of patients

☐ Of all patients with type 2 diabetes mellitus who use BOLUS insulin

☐ None

☐ 1-10% of patients

☐ 11-20% of patients

☐ 21-30% of patients

☐ 31-40% of patients

☐ 41-50% of patients

☐ 51-60% of patients

☐ 61-70% of patients

☐ 71-80% of patients

☐ 81-90% of patients

☐ 91-100% of patients

9. In your opinion, in the past 30 days, what are the most common factors that caused your patients with type 1 or 2 diabetes mellitus to mistime BOLUS insulin doses? (Only show for respondents who have indicated that their patients mistime doses)

Please select all that apply

☐ They forgot

☐ They were too busy/distracted

☐ They find it too complicated and burdensome

☐ They were out of their normal routine

☐ They did not want to dose in front of others

☐ They weren't sure how much insulin to take due to unfamiliar food

☐ They had an unexpected meal or ate earlier or later than expected

☐ They couldn't remember when they last took a dose

☐ They did not measure their blood glucose

☐ They wanted to avoid their blood sugar getting too low

☐ They were concerned about weight gain

☐ They wanted to save on the cost of insulin

☐ Other (please specify) [Open Text Field]

10. **Please rank in order from 1-3 the most common reasons from those you have selected as the reasons why your patients mistimed BOLUS insulin doses.**

    Please select your top three most common reasons and rank them in order, using the drag and drop function below.

    [Ranking exercise repeating all responses selected in Q9]

11. **In the past 30 days, what proportion of your patients with type 1 or 2 diabetes mellitus do you think mistimed BASAL insulin doses?**

    Please select only one for each column

    ☐ Of all patients with <u>type 1</u> diabetes mellitus

    - ☐ None
    - ☐ 1-10% of patients
    - ☐ 11-20% of patients
    - ☐ 21-30% of patients
    - ☐ 31-40% of patients
    - ☐ 41-50% of patients
    - ☐ 51-60% of patients
    - ☐ 61-70% of patients
    - ☐ 71-80% of patients
    - ☐ 81-90% of patients
    - ☐ 91-100% of patients

    ☐ Of all patients with type 2 diabetes mellitus

    - ☐ None
    - ☐ 1-10% of patients
    - ☐ 11-20% of patients
    - ☐ 21-30% of patients
    - ☐ 31-40% of patients
    - ☐ 41-50% of patients
    - ☐ 51-60% of patients

☐ 61-70% of patients

☐ 71-80% of patients

☐ 81-90% of patients

☐ 91-100% of patients

12. In your opinion, in the past 30 days, what are the most common factors that caused your patients with type 1 or 2 diabetes mellitus to mistime BASAL insulin doses? (Only show for respondents who have indicated that their patients mistime doses)

    Please select all that apply

    ☐ They forgot

    ☐ They were too busy/distracted

    ☐ They find it too complicated and burdensome

    ☐ They were out of their normal routine

    ☐ They did not want to dose in front of others

    ☐ They weren't sure how much insulin to take due to unfamiliar food

    ☐ They had an unexpected meal or ate earlier or later than expected

    ☐ They couldn't remember when they last took a dose

    ☐ They did not measure their blood glucose

    ☐ They wanted to avoid their blood sugar getting too low

    ☐ They were concerned about weight gain

    ☐ They wanted to save on the cost of insulin

    ☐ Other (please specify) [Open Text Field]

13. Please rank in order from 1-3 the most common reasons from those you have selected as the reasons why your patients mistimed BASAL insulin doses.

    Please select your top three most common reasons and rank them in order, using the drag and drop function below.

    [Ranking exercise repeating all responses selected in Q12]

MISCALCULATED INSULIN DOSES

The following questions ask you about any insulin doses your patients may have miscalculated. By miscalculating a dose, we mean any time a patient has unintentionally taken too little insulin.

When answering the questions in this section, please refer only to adult patients with T1DM and T2DM using insulin pens.

14. In the past 30 days, what proportion of your patients with type 1 or 2 diabetes mellitus do you think miscalculated BOLUS insulin doses?

    Please select only one for each column

    ☐ Of all patients with type 1 diabetes mellitus who use BOLUS insulin

    - ☐ None
    - ☐ 1-10% of patients
    - ☐ 11-20% of patients
    - ☐ 21-30% of patients
    - ☐ 31-40% of patients
    - ☐ 41-50% of patients
    - ☐ 51-60% of patients
    - ☐ 61-70% of patients
    - ☐ 71-80% of patients
    - ☐ 81-90% of patients
    - ☐ 91-100% of patients

    ☐ Of all patients with type 2 diabetes mellitus who use BOLUS insulin

    - ☐ None
    - ☐ 1-10% of patients
    - ☐ 11-20% of patients
    - ☐ 21-30% of patients
    - ☐ 31-40% of patients
    - ☐ 41-50% of patients
    - ☐ 51-60% of patients
    - ☐ 61-70% of patients
    - ☐ 71-80% of patients
    - ☐ 81-90% of patients
    - ☐ 91-100% of patients

15. In your opinion, in the past 30 days, what are the main factors that caused your patients with type 1 or 2 diabetes mellitus to miscalculate BOLUS insulin doses? (Only show for respondents who have indicated that their patients miscalculate doses)

Please select all that apply

☐ They find it too complicated and burdensome

☐ They were out of their normal routine

☐ They did not want to dose in front of others

☐ They weren't sure how much insulin to take (e.g., sick, exercising, unfamiliar food, insulin plan was not available)

☐ They couldn't remember when they last took a dose

☐ They did not measure their blood glucose

☐ They wanted to avoid their blood sugar getting too low

☐ They were trying to save on the cost of insulin

☐ Other (please specify) [Open Text Field]

16. **Please rank in order from 1-3 the most common reasons from those you have selected as the reasons why your patients miscalculated a BOLUS insulin doses.**

    Please select your top three most common reasons and rank them in order, using the drag and drop function below.

    [Ranking exercise from 1 to 3 repeating all responses selected in Q15]

17. **In the past 30 days, what proportion of your patients with type 1 or 2 diabetes mellitus do you think miscalculated BASAL insulin doses?**

    Please select only one for each column

    ☐ Of all patients with <u>type 1</u> diabetes mellitus

    - ☐ None
    - ☐ 1-10% of patients
    - ☐ 11-20% of patients
    - ☐ 21-30% of patients
    - ☐ 31-40% of patients
    - ☐ 41-50% of patients
    - ☐ 51-60% of patients
    - ☐ 61-70% of patients
    - ☐ 71-80% of patients
    - ☐ 81-90% of patients
    - ☐ 91-100% of patients

☐ Of all patients with type 2 diabetes mellitus

☐ None

☐ 1-10% of patients

☐ 11-20% of patients

☐ 21-30% of patients

☐ 31-40% of patients

☐ 41-50% of patients

☐ 51-60% of patients

☐ 61-70% of patients

☐ 71-80% of patients

☐ 81-90% of patients

☐ 91-100% of patients

18. In your opinion, in the past 30 days, what are the main factors that caused your patients with type 1 or 2 diabetes mellitus to miscalculate BASAL insulin doses? (Only show for respondents who have indicated that their patients miscalculate doses)

Please select all that apply

☐ They find it too complicated and burdensome

☐ They were out of their normal routine

☐ They did not want to dose in front of others

☐ They weren't sure how much insulin to take (e.g., sick, exercising, unfamiliar food, insulin plan was not available)

☐ They couldn't remember when they last took a dose

☐ They did not measure their blood glucose

☐ They wanted to avoid their blood sugar getting too low

☐ They were trying to save on the cost of insulin

☐ Other (please specify) [Open Text Field]

19. Please rank in order from 1-3 the most common reasons from those you have selected as the reasons why your patients miscalculated a BASAL insulin doses.

Please select your top three most common reasons and rank them in order, using the drag and drop function below.

[Ranking exercise from 1 to 3 repeating all responses selected in Q18]

Insulin Dosing Issues and Solutions

When answering the questions in this section, please refer only to adult patients with T1DM and T2DM using insulin pens (excluding pre-mix).

1. What proportion of your patients do you believe have their glucose levels adequately under control, according to your local guidelines? Filter question based on response to Q 6 in the section “current treatment and routines” – if someone replies there ‘None’ T1DM or T2DM patients do NOT show them the T1DM or T2DM option here as relevant

   Please select only one/ please select only one for each column ***(instructions shown will be based on Q6 selection)***

   ☐ Of all patients with type 1 diabetes mellitus

   - ☐ None
   - ☐ 1-10% of patients
   - ☐ 11-20% of patients
   - ☐ 21-30% of patients
   - ☐ 31-40% of patients
   - ☐ 41-50% of patients
   - ☐ 51-60% of patients
   - ☐ 61-70% of patients
   - ☐ 71-80% of patients
   - ☐ 81-90% of patients
   - ☐ 91-100% of patients

   Please select only one

   ☐ Of all patients with type 2 diabetes mellitus

   - ☐ None
   - ☐ 1-10% of patients
   - ☐ 11-20% of patients
   - ☐ 21-30% of patients
   - ☐ 31-40% of patients
   - ☐ 41-50% of patients
   - ☐ 51-60% of patients

☐ 61-70% of patients

☐ 71-80% of patients

☐ 81-90% of patients

☐ 91-100% of patients

2. What proportion of your patients do you believe need to have their treatment escalated to have their glucose levels adequately under control? (e.g., dose increase/adding combination therapy) Filter question based on response to Q 6 in the section "current treatment and routines" – if someone replies there 'None' T1DM or T2DM patients do NOT show them the T1DM or T2DM option here as relevant

   Please select only one/ please select only one for each column ***(instructions shown will be based on Q6 selection)***

   ☐ Of all patients with type 1 diabetes mellitus

   ☐ None

   ☐ 1-10% of patients

   ☐ 11-20% of patients

   ☐ 21-30% of patients

   ☐ 31-40% of patients

   ☐ 41-50% of patients

   ☐ 51-60% of patients

   ☐ 61-70% of patients

   ☐ 71-80% of patients

   ☐ 81-90% of patients

   ☐ 91-100% of patients

   ☐ Of all patients with type 2 diabetes mellitus

   ☐ None

   ☐ 1-10% of patients

   ☐ 11-20% of patients

   ☐ 21-30% of patients

   ☐ 31-40% of patients

   ☐ 41-50% of patients

   ☐ 51-60% of patients

☐ 61-70% of patients

☐ 71-80% of patients

☐ 81-90% of patients

☐ 91-100% of patients

3. **How much do you agree that titration could be done more quickly to get a better result for patients using BASAL or BOLUS insulin?**

   Please select only one

   ☐ Strongly agree

   ☐ Agree

   ☐ Neutral

   ☐ Disagree

   ☐ Strongly disagree

4. **As a treating healthcare professional, what could help you to more quickly titrate patients using BOLUS insulin to a more optimal dose?**

   Please select all that apply

   ☐ More objective and reliable data on insulin dosing

   ☐ More objective data on blood glucose levels

   ☐ Patient data portals where I can readily see their insulin and glucose data

   ☐ More time with patients

   ☐ Lower patient load

   ☐ More patient education

   ☐ Other (please specify) [Open Text Field]

5. **As a treating healthcare professional, what could help you to more quickly titrate patients using BASAL insulin to a more optimal dose?**

   Please select all that apply☐ More objective and reliable data on insulin dosing

   ☐ More objective data on blood glucose levels

   ☐ Patient data portals where I can readily see their insulin and glucose data

   ☐ More time with patients

   ☐ Lower patient load

   ☐ More patient education

☐ Other (please specify) [Open Text Field]

6. For what proportion of your patients do you believe managing insulin dosing is complicated and/or burdensome? Filter question based on response to Q 6 in the section "current treatment and routines" – if someone replies there 'None' T1DM or T2DM patients do NOT show them the T1DM or T2DM option here as relevant

   Please select only one/ please select only one for each column ***(instructions shown will be based on Q6 selection)***

   ☐ Of all patients with <u>type 1</u> diabetes mellitus

   - ☐ None
   - ☐ 1-10% of patients
   - ☐ 11-20% of patients
   - ☐ 21-30% of patients
   - ☐ 31-40% of patients
   - ☐ 41-50% of patients
   - ☐ 51-60% of patients
   - ☐ 61-70% of patients
   - ☐ 71-80% of patients
   - ☐ 81-90% of patients
   - ☐ 91-100% of patients

   ☐ Of all patients with <u>type 2</u> diabetes mellitus

   - ☐ None
   - ☐ 1-10% of patients
   - ☐ 11-20% of patients
   - ☐ 21-30% of patients
   - ☐ 31-40% of patients
   - ☐ 41-50% of patients
   - ☐ 51-60% of patients
   - ☐ 61-70% of patients
   - ☐ 71-80% of patients
   - ☐ 81-90% of patients
   - ☐ 91-100% of patients

7. **What proportion of your patients do you believe fully adhere to their prescribed mealtime/BOLUS insulin routine? Filter question based on response to Q 6 in the section “current treatment and routines” – if someone replies there ‘None’ T1DM or T2DM patients do NOT show them the T1DM or T2DM option here as relevant**

   Please select only one/ please select only one for each column ***(instructions shown will be based on Q6 selection)***

   ☐ Of all patients with type 1 diabetes mellitus administering BOLUS doses

   - ☐ None
   - ☐ 1-10% of patients
   - ☐ 11-20% of patients
   - ☐ 21-30% of patients
   - ☐ 31-40% of patients
   - ☐ 41-50% of patients
   - ☐ 51-60% of patients
   - ☐ 61-70% of patients
   - ☐ 71-80% of patients
   - ☐ 81-90% of patients
   - ☐ 91-100% of patients

   ☐ Of all patients with type 2 diabetes mellitus administering BOLUS doses

   - ☐ None
   - ☐ 1-10% of patients
   - ☐ 11-20% of patients
   - ☐ 21-30% of patients
   - ☐ 31-40% of patients
   - ☐ 41-50% of patients
   - ☐ 51-60% of patients
   - ☐ 61-70% of patients
   - ☐ 71-80% of patients
   - ☐ 81-90% of patients
   - ☐ 91-100% of patients

8. What proportion of your patients do you believe fully adhere to their prescribed BASAL insulin routine? Filter question based on response to Q 6 in the section “current treatment and routines” – if someone replies there ‘None’ T1DM or T2DM patients do NOT show them the T1DM or T2DM option here as relevant

   Please select only one/ please select only one for each column ***(instructions shown will be based on Q6 selection)***

   ☐ Of all patients with <u>type 1</u> diabetes mellitus

   ☐ None

   ☐ 1-10% of patients

   ☐ 11-20% of patients

   ☐ 21-30% of patients

   ☐ 31-40% of patients

   ☐ 41-50% of patients

   ☐ 51-60% of patients

   ☐ 61-70% of patients

   ☐ 71-80% of patients

   ☐ 81-90% of patients

   ☐ 91-100% of patients

   ☐ Of all patients with <u>type 2</u> diabetes mellitus

   ☐ None

   ☐ 1-10% of patients

   ☐ 11-20% of patients

   ☐ 21-30% of patients

   ☐ 31-40% ofNot patients

   ☐ 41-50% of patients

   ☐ 51-60% of patients

   ☐ 61-70% of patients

   ☐ 71-80% of patients

   ☐ 81-90% of patients

   ☐ 91-100% of patients Not

9. In your opinion, what proportion of your patients with type 1 or 2 diabetes mellitus who skip, miss, or forget their BOLUS insulin doses report this to you?

Filter question based on response to Q 6 in the section “current treatment and routines” – if someone replies there ‘None’ T1DM or T2DM patients do NOT show them the T1DM or T2DM option here as relevant

- ☐ Type 1 patients

  Please select one for each column

  - ☐ None
  - ☐ 1-10% of patients
  - ☐ 11-20% of patients
  - ☐ 21-30% of patients
  - ☐ 31-40% of patients
  - ☐ 41-50% of patients
  - ☐ 51-60% of patients
  - ☐ 61-70% of patients
  - ☐ 71-80% of patients
  - ☐ 81-90% of patients
  - ☐ 91-100% of patients

- ☐ Type 2 patients

  - ☐ None
  - ☐ 1-10% of patients
  - ☐ 11-20% of patients
  - ☐ 21-30% of patients
  - ☐ 31-40% of patients
  - ☐ 41-50% of patients
  - ☐ 51-60% of patients
  - ☐ 61-70% of patients
  - ☐ 71-80% of patients
  - ☐ 81-90% of patients
  - ☐ 91-100% of patients

10. **In your opinion, for what reasons do patients with type 1 or 2 diabetes mellitus not report skipping, missing, or forgetting their BOLUS insulin doses to you?**

Please select all that apply:

☐ Patients are unaware that they are not adherent

☐ Patients do not want to disclose to you that they are not taking the regimen as prescribed

☐ Patients do not realize the impacts of skipping/mistiming or miscalculating doses

☐ Patients are not concerned about the impacts of skipping/mistiming or miscalculating doses

☐ Patients do not record or document their insulin doses

☐ Other (please specify) [Open Text Field]

11. **How confident are you in initiating insulin with patients?**

Please select only one

☐ Very confident

☐ Somewhat confident

☐ Neither confident nor in doubt

☐ Not confident

12. **How confident are you in titrating insulin with patients?**

Please select only one

☐ Very confident

☐ Somewhat confident

☐ Neither confident nor in doubt

☐ Not confident

13. **Rate the difficulty you face with patients regarding the following aspects. Please answer first about initiating insulin and then about titrating insulin.**

Please rate each statement from extremely difficult to not at all difficult

	Extremely difficult	Moderately difficult	Slightly Difficult	Neutral	Not at all difficult
Initiating Insulin					
Lack of objective blood glucose data to base my decisions on	☐	☐	☐	☐	☐
Uncertain how well my patients will follow an insulin regimen	☐	☐	☐	☐	☐

	Extremely difficult	Moderately difficult	Slightly Difficult	Neutral	Not at all difficult
Patient considerations (e.g., work schedule, lifestyle) that may interfere with their ability to follow an insulin regimen	☐	☐	☐	☐	☐
Motivating patients to adhere to their prescribed insulin dose	☐	☐	☐	☐	☐
Getting patients to understand the impact of missing/mistiming/ miscalculating doses	☐	☐	☐	☐	☐
Balancing efficacy of the insulin vs. avoiding hypoglycemic episodes	☐	☐	☐	☐	☐
Amount of time it takes for me to figure out the best approach for a particular patient	☐	☐	☐	☐	☐
Amount of time it takes for me and my staff to convince and educate the patient on their insulin regimen	☐	☐	☐	☐	☐
Titrating Insulin					
Trusting what patients are reporting about their insulin adherence	☐	☐	☐	☐	☐
Lack of objective insulin data to base my decisions on	☐	☐	☐	☐	☐
Lack of objective blood glucose data to base my decisions on	☐	☐	☐	☐	☐
Uncertain how well my patients follow their insulin regimen	☐	☐	☐	☐	☐
Motivating patients to adhere to their prescribed insulin dose	☐	☐	☐	☐	☐
Getting patients to understand the impact of missing/mistiming/ miscalculating doses	☐	☐	☐	☐	☐
Balancing efficacy of the insulin vs. avoiding hypoglycemic episodes	☐	☐	☐	☐	☐
Amount of time it takes for me to figure out the best approach for a particular patient	☐	☐	☐	☐	☐
Amount of time it takes for me and my staff to follow my patients' progress and recommend dose increases	☐	☐	☐	☐	☐

14. How much do you think the following things would help your patients to optimize their insulin dosing (e.g., forgetting less doses, taking more doses on time and not making calculation errors)?

Please rate each option from very helpful to not at all helpful

	Very helpful	Somewhat helpful	Slightly helpful	Neutral	Not at all helpful
Real-time insulin dosing calculation guidance	☐	☐	☐	☐	☐
Dosing reminders in a mobile application	☐	☐	☐	☐	☐
Automated food diary/carb counting in a mobile application	☐	☐	☐	☐	☐
A device that automatically records insulin doses and timing	☐	☐	☐	☐	☐
A device that automatically records glucose measurements	☐	☐	☐	☐	☐
Having a patient’s insulin and glucose data combined in one place	☐	☐	☐	☐	☐
Having near real-time feedback on how a patient’s insulin dosing impacts their glucose levels	☐	☐	☐	☐	☐
Having more time with my patients to discuss their insulin dosing routine	☐	☐	☐	☐	☐
More meaningful conversations with my patients about their insulin dosing routine	☐	☐	☐	☐	☐

15. Are you already prescribing smart/connected insulin pens or caps in your practice?

Please select only one

☐ Yes

☐ No

☐ Not currently, but thinking about it

Thank you, you have completed the survey!
