# Supplementary material for: Understanding suboptimal insulin use in type 1 and 2 diabetes: a cross-sectional survey of healthcare providers who treat people with diabetes
Source: BMC Prim Care. 2024 Apr 22;25:124. doi: 10.1186/s12875-024-02390-9 (PMC11034124; doi:10.1186/s12875-024-02390-9)
Supplement: Supplementary file 2 — Supplementary Material 2. [file 12875_2024_2390_MOESM2_ESM.docx]

# Additional File 2

**Supplementary Table 1. General Practitioners’ and Specialists’ Estimated Proportion of PwD Who Missed/Skipped Insulin Doses, Not Due to Missing a Meal**

| HCP Estimates of PwD Dosing Behavior | Total Number of HCPs (N=640) | | | | |
| --- | --- | --- | --- | --- | --- |
|  | General Practitioners  (N=320) | | Specialists  (N=320) | | |
| n (%) | Bolus | Basal | Bolus | Basal | |
| Type 1 Diabetes | | | | | |
| None | 60 (18.8) | 87 (27.2) | 27 (8.4) | | 55 (17.2) |
| 1–10% | 136 (42.5%) | 142 (44.4%) | 113 (35.3%) | | 146 (45.6%) |
| 11–20% | 55 (17.2%) | 45 (14.1%) | 73 (22.8%) | | 61 (19.1%) |
| 21–30% | 26 (8.1%) | 23 (7.2%) | 41 (12.8%) | | 22 (6.9%) |
| 31–40% | 16 (5.0%) | 6 (1.9%) | 18 (5.6%) | | 15 (4.7%) |
| 41–50% | 11 (3.4%) | 4 (1.3%) | 15 (4.7%) | | 9 (2.8%) |
| 51–60% | 3 (0.9%) | 6 (1.9%) | 11 (3.4%) | | 4 (1.3%) |
| 61–70% | 9 (2.8%) | 3 (0.9%) | 6 (1.9%) | | 2 (0.6%) |
| 71–80% | 2 (0.6%) | 3 (0.9%) | 7 (2.2%) | | 4 (1.3%) |
| 81–90% | 1 (0.3%) | No data | 7 (2.2%) | | 2 (0.6%) |
| 91–100% | 1 (0.3%) | 1 (0.3%) | 2 (0.6%) | | No data |
| Type 2 Diabetes | | | | | |
| None | 35 (10.9) | 39 (12.2) | 8 (2.5) | | 20 (6.3) |
| 1–10% | 101 (31.6%) | 125 (39.1%) | 77 (24.1%) | | 115 (35.9%) |
| 11–20% | 68 (21.3%) | 66 (20.6%) | 84 (26.3%) | | 82 (25.6%) |
| 21–30% | 43 (13.4%) | 36 (11.3%) | 58 (18.1%) | | 37 (11.6%) |
| 31–40% | 25 (7.8%) | 18 (5.6%) | 25 (7.8%) | | 25 (7.8%) |
| 41–50% | 14 (4.4%) | 15 (4.7%) | 23 (7.2%) | | 16 (5.0%) |
| 51–60% | 10 (3.1%) | 8 (2.5%) | 10 (3.1%) | | 14 (4.4%) |
| 61–70% | 10 (3.1%) | 4 (1.3%) | 6 (1.9%) | | 3 (0.9%) |
| 71–80% | 10 (3.1%) | 3 (0.9%) | 12 (3.8%) | | 2 (0.6%) |
| 81–90% | 2 (0.6%) | 4 (1.3%) | 14 (4.4%) | | 5 (1.6%) |
| 91–100% | 2 (0.6%) | 2 (0.6%) | 3 (0.9%) | | 1 (0.3%) |

HCP = healthcare provider; n = number of HCPs in the specified category; N = number of HCPs in analysis; PwD = people with diabetes.

HCP estimates were for their impressions of PwD with suboptimal dosing in the past 30 days not due to skipping a meal.

**Supplementary Table 2. HCP Estimated Proportion of PwD Who Mistimed Insulin Doses, Not Due to Missing a Meal**

| HCP Estimates of PwD Adherence | Total Number of HCPs (N=640) | | |
| --- | --- | --- | --- |
| n (%) | Bolus | | Basal |
| Type 1 Diabetes | | | |
| None | 71 (11.1%) | 119 (18.6%) | |
| 1–10% | 218 (34.1%) | 284 (44.4%) | |
| 11–20% | 144 (22.5%) | 107 (16.7%) | |
| 21–30% | 80 (12.5%) | 56 (8.8%) | |
| 31–40% | 46 (7.2%) | 34 (5.3%) | |
| 41–50% | 29 (4.5%) | 12 (1.9%) | |
| 51–60% | 19 (3.0%) | 12 (1.9%) | |
| 61–70% | 14 (2.2%) | 8 (1.3%) | |
| 71–80% | 10 (1.6%) | 4 (0.6%) | |
| 81–90% | 5 (0.8%) | 3 (0.5%) | |
| 91–100% | 4 (0.6%) | 1 (0.2%) | |
| Type 2 Diabetes | | | |
| None | 44 (6.9%) | 70 (10.9%) | |
| 1–10% | 164 (25.6%) | 225 (35.2%) | |
| 11–20% | 151 (23.6%) | 145 (22.7%) | |
| 21–30% | 102 (15.9%) | 81 (12.7%) | |
| 31–40% | 59 (9.2%) | 42 (6.6%) | |
| 41–50% | 40 (6.3%) | 26 (4.1%) | |
| 51–60% | 33 (5.2%) | 22 (3.4%) | |
| 61–70% | 20 (3.1%) | 7 (1.1%) | |
| 71–80% | 13 (2.0%) | 10 (1.6%) | |
| 81–90% | 10 (1.6%) | 10 (1.6%) | |
| 91–100% | 4 (0.6%) | 2 (0.3%) | |

HCP = healthcare provider; PwD = people with diabetes; T1D = type 1 diabetes; T2D = type 2 diabetes.

HCP estimates were for their impression of the proportion of PwD with suboptimal dosing in the past 30 days not due to skipping a meal.

**Supplementary Table 3. HCP Estimated Proportions of PwD who Missed/Skipped Their Bolus Insulin Doses Due to Skipping a Meal in the Past 30 Days**

| HCP Estimates of PwD Adherence | Total Number of HCPs (N=640) | | |
| --- | --- | --- | --- |
| n (%) | Type 1 Diabetes Bolus | | Type 2 Diabetes  Bolus |
| None | 80 (12.5%) | 33 (5.2%) | |
| 1–10% | 186 (29.1%) | 137 (21.4%) | |
| 11–20% | 145 (22.7%) | 153 (23.9%) | |
| 21–30% | 84 (13.1%) | 117 (18.3%) | |
| 31–40% | 42 (6.6%) | 65 (10.2%) | |
| 41–50% | 27 (4.2%) | 45 (7.0%) | |
| 51–60% | 17 (2.7%) | 25 (3.9%) | |
| 61–70% | 16 (2.5%) | 28 (4.4%) | |
| 71–80% | 20 (3.1%) | 14 (2.2%) | |
| 81–90% | 13 (2.0%) | 15 (2.3%) | |
| 91–100% | 10 (1.6%) | 8 (1.3%) | |

HCP = healthcare provider; PwD = people with diabetes; T1D = type 1 diabetes; T2D = type 2 diabetes

HCP estimates were for their impression of the proportion of PwD with suboptimal dosing in the past 30 days due to skipping a meal.

**Supplementary Table 4. HCP Estimated Proportion of PwD who Miscalculated Their Insulin**

| HCP Estimates of PwD Adherence | Total Number of HCPs (N=640) | | |
| --- | --- | --- | --- |
| n (%) | Bolus | | Basal |
| Type 1 Diabetes | | | |
| None | 72 (11.3%) | 153 (23.9%) | |
| 1–10% | 220 (34.4%) | 252 (39.4%) | |
| 11–20% | 140 (21.9%) | 118 (18.4%) | |
| 21–30% | 88 (13.8%) | 53 (8.3%) | |
| 31–40% | 41 (6.4%) | 30 (4.7%) | |
| 41–50% | 22 (3.4%) | 13 (2.0%) | |
| 51–60% | 21 (3.3%) | 8 (1.3%) | |
| 61–70% | 19 (3.0%) | 5 (0.8%) | |
| 71–80% | 7 (1.1%) | 2 (0.3%) | |
| 81–90% | 6 (0.9%) | 5 (0.8%) | |
| 91–100% | 4 (0.6%) | 1 (0.2%) | |
| Type 2 Diabetes | | | |
| None | 37 (5.8%) | 96 (15.0%) | |
| 1–10% | 172 (26.9%) | 219 (34.2%) | |
| 11–20% | 129 (20.2%) | 129 (20.2%) | |
| 21–30% | 117 (18.3%) | 89 (13.9%) | |
| 31–40% | 70 (10.9%) | 39 (6.1%) | |
| 41–50% | 38 (5.9%) | 19 (3.0%) | |
| 51–60% | 24 (3.8%) | 20 (3.1%) | |
| 61–70% | 14 (2.2%) | 13 (2.0%) | |
| 71–80% | 25 (3.9%) | 9 (1.4%) | |
| 81–90% | 9 (1.4%) | 5 (0.8%) | |
| 91–100% | 5 (0.8%) | 2 (0.3%) | |

HCP = healthcare provider; PwD = people with diabetes; T1D = type 1 diabetes; T2D = type 2 diabetes.

HCP estimates were for their impression of the proportion of PwD with suboptimal dosing in the past 30 days.

**Supplementary Table 5. Proportion of PwD who HCPs Estimate Found Insulin Dosing/Management Complicated and/or Burdensome**

|  | PwD Categories | Total  (N=640) |
| --- | --- | --- |
| T1D |  |  |
|  | None | 25 (3.9) |
|  | 1–10% | 153 (23.9%) |
|  | 11–20% | 146 (22.8%) |
|  | 21–30% | 108 (16.9%) |
|  | 31–40% | 50 (7.8%) |
|  | 41–50% | 29 (4.5%) |
|  | 51–60% | 38 (5.9%) |
|  | 61–70% | 28 (4.4%) |
|  | 71–80% | 18 (2.8%) |
|  | 81–90% | 13 (2.0%) |
|  | 91–100% | 11 (1.7%) |
|  | Missing* | 21 (3.3%) |
| T2D |  |  |
|  | None | 15 (2.3) |
|  | 1–10% | 90 (14.1%) |
|  | 11–20% | 119 (18.6%) |
|  | 21–30% | 119 (18.6%) |
|  | 31–40% | 85 (13.3%) |
|  | 41–50% | 62 (9.7%) |
|  | 51–60% | 51 (8.0%) |
|  | 61–70% | 33 (5.2%) |
|  | 71–80% | 33 (5.2%) |
|  | 81–90% | 16 (2.5%) |
|  | 91–100% | 13 (2.0%) |
|  | Missing* | 4 (0.6%) |

HCP = healthcare provider; N = number of HCPs in the study population; PwD = people with diabetes; T1D = type 1 diabetes; T2D = type 2 diabetes.

*Missing counts are participants who answered “none” to Survey Question 6, which asked “approximately how many adult patients on insulin pens are you currently treating?”

**Supplementary Table 6. Proportion of PwD that HCPs Felt Fully Adhered to Their Prescribed Insulin Dosing Routine**

| HCP Estimates of PwD Adherence | Total Number of HCPs (N=640) | | |
| --- | --- | --- | --- |
| n (%) | Bolus | | Basal |
| Type 1 Diabetes | | | |
| None | 3 (0.5%) | 5 (0.8%) | |
| 1–10% | 53 (8.3%) | 37 (5.8%) | |
| 11–20% | 53 (8.3%) | 35 (5.5%) | |
| 21–30% | 49 (7.7%) | 30 (4.7%) | |
| 31–40% | 43 (6.7%) | 25 (3.9%) | |
| 41–50% | 36 (5.6%) | 30 (4.7%) | |
| 51–60% | 64 (10.0%) | 43 (6.7%) | |
| 61–70% | 68 (10.6%) | 49 (7.7%) | |
| 71–80% | 112 (17.5%) | 117 (18.3%) | |
| 81–90% | 101 (15.8%) | 156 (24.4%) | |
| 91–100% | 37 (5.8%) | 92 (14.4%) | |
| Missing* | 21 (3.3%) | 21 (3.3%) | |
| Type 2 Diabetes | | | |
| None | 6 (0.9%) | 8 (1.3%) | |
| 1–10% | 42 (6.6%) | 28 (4.4%) | |
| 11–20% | 74 (11.6%) | 47 (7.3%) | |
| 21–30% | 66 (10.3%) | 36 (5.6%) | |
| 31–40% | 52 (8.1%) | 38 (5.9%) | |
| 41–50% | 59 (9.2%) | 52 (8.1%) | |
| 51–60% | 88 (13.8%) | 57 (8.9%) | |
| 61–70% | 110 (17.2%) | 110 (17.2%) | |
| 71–80% | 81 (12.7%) | 128 (20.0%) | |
| 81–90% | 47 (7.3%) | 102 (15.9%) | |
| 91–100% | 11 (1.7%) | 30 (4.7%) | |
| Missing* | 4 (0.6%) | 4 (0.6%) | |

HCP = healthcare provider; n = number of HCPs in the specified category; N = number of HCPs in analysis population; PwD = people with diabetes.

* Missing counts are participants who answered “none” to Survey Question 6, which asked “approximately how many adult patients on insulin pens are you currently treating?”
